# Pathological Features and Neuroinflammatory Mechanisms of SARS-CoV-2 in the Brain and Potential Therapeutic Approaches

**DOI:** 10.3390/biom12070971

**Published:** 2022-07-11

**Authors:** Aisha Sodagar, Rasab Javed, Hira Tahir, Saiful Izwan Abd Razak, Muhammad Shakir, Muhammad Naeem, Abdul Halim Abdul Yusof, Suresh Sagadevan, Abu Hazafa, Jalal Uddin, Ajmal Khan, Ahmed Al-Harrasi

**Affiliations:** 1 Department of Botany, Faculty of Sciences, University of Agriculture, Faisalabad 38040, Pakistan; aishasodagar17@gmail.com; 2 Institute of Microbiology, University of Agriculture, Faisalabad 38040, Pakistan; rasabjaved9@gmail.com; 3 Department of Botany, Government College Women University Faisalabad, Faisalabad 38000, Pakistan; hiratahir14166@gmail.com; 4 Bioinspired Device and Tissue Engineering Research Group, School of Biomedical Engineering and Health Sciences, Faculty of Engineering, Universiti Teknologi Malaysia, Johor Bahru 81310, Johor, Malaysia; saifulizwan@utm.my; 5 Sports Innovation & Technology Centre, Institute of Human Centred Engineering, Universiti Teknologi Malaysia, Johor Bahru 81310, Johor, Malaysia; 6 School of Life Sciences, Northeast Normal University, Changchun 130024, China; shakirkhan1418@gmail.com; 7 College of Life Science, Hebei Normal University, Shijiazhuang 050024, China; naeemsaleem413@gmail.com; 8 School of Chemical and Energy Engineering, Faculty of Engineering, Universiti Teknologi Malaysia, Johor Bahru 81310, Johor, Malaysia; halimy@utm.my; 9 Nanotechnology & Catalysis Research Centre, University of Malaya, Kuala Lumpur 50603, Kuala Lumpur, Malaysia; drsureshnano@gmail.com; 10 Department of Biochemistry, Faculty of Sciences, University of Agriculture, Faisalabad 38040, Pakistan; 11 Department of Pharmaceutical Chemistry, College of Pharmacy, King Khalid University, Abha 62529, Saudi Arabia; jalaluddinamin@gmail.com; 12 Natural and Medical Sciences Research Center, University of Nizwa, Birkat Al Mauz, Nizwa 616, Oman

**Keywords:** neurological symptoms, SARS-CoV-2, neuroinflammation, pathological feathers, cytokines, brain, COVID-19, olfactory bulb, therapeutic approaches

## Abstract

The number of deaths has been increased due to COVID-19 infections and uncertain neurological complications associated with the central nervous system. Post-infections and neurological manifestations in neuronal tissues caused by COVID-19 are still unknown and there is a need to explore how brainstorming promoted congenital impairment, dementia, and Alzheimer’s disease. SARS-CoV-2 neuro-invasion studies in vivo are still rare, despite the fact that other beta-coronaviruses have shown similar properties. Neural (olfactory or vagal) and hematogenous (crossing the blood–brain barrier) pathways have been hypothesized in light of new evidence showing the existence of SARS-CoV-2 host cell entry receptors into the specific components of human nerve and vascular tissue. Spike proteins are the primary key and structural component of the COVID-19 that promotes the infection into brain cells. Neurological manifestations and serious neurodegeneration occur through the binding of spike proteins to ACE2 receptor. The emerging evidence reported that, due to the high rate in the immediate wake of viral infection, the olfactory bulb, thalamus, and brain stem are intensely infected through a trans-synaptic transfer of the virus. It also instructs the release of chemokines, cytokines, and inflammatory signals immensely to the blood–brain barrier and infects the astrocytes, which causes neuroinflammation and neuron death; and this induction of excessive inflammation and immune response developed in more neurodegeneration complications. The present review revealed the pathophysiological effects, molecular, and cellular mechanisms of possible entry routes into the brain, pathogenicity of autoantibodies and emerging immunotherapies against COVID-19.

## 1. Introduction

Severe acute respiratory syndrome (SARS), a disease caused by Coronavirus-2 (CoV-2), belongs to β-coronaviruses, is respiratory in nature with pulmonary effects [1,2,3], but also has a major impact on the central nervous system, as well as peripheral nervous system impairment [4]. Coronaviruses have feasibly different way enroute to the brain, these viruses cross the blood-CSF barrier or cross the blood–brain barrier. A virus may use immune cells that have the ability to cross the barrier naturally and via endothelial cells of the blood–brain barrier. Coronavirus usually replicates in peripheral blood mononuclear cells (PBMCs), but its ability to imitate is still unknown [5]. Consistent neurological impairment is scrutinized in patients infected with a deadly virus, and this fact was verified by studying collected works and case inquiries exactly after the onset of this pandemic disease. Clear evidence of an impaired nervous system of coronavirus-2 infected patients was perceived after viral RNA detection in their cerebrospinal fluid. Evidence demonstrated that the coronavirus infected astrocytes to cause neuro-infection by endocytosis [1,6] via NRP1 receptors and due to proliferated expression of BSG mRNA [1].

Three-dimensional structure of spike protein (S); the receptor-binding domain had massive similarities by sharing a high percentage of nucleotide sequence between coronavirus SARS-CoV and SARS-CoV-2, which supports the theory of viral infection via ACE2 receptor that is expressed in brain and SARS-CoV-2 with greater affinity [3,6]. However, the expression of ACE2 with other cofactors (TMPRSS2 -transmembrane serine protease-2 and Neuropilin-1) is still indecisive due to low concentrations of mRNA for ACE2 receptors in COVID-19 influenced patients [7].

The septic astrocytes show off incredible changes in the use of metabolites to excite the neurons to the abnormal composition of neurotransmitters, soluble factors, and increased secretory phenotype that possibly became the reason for neuronal death. This is one of the significant reasons for neuropsychological symptoms in COVID-19 patients, but we still lack knowledge about the severity of its infection in brain cells [1]. SARS-CoV-2 can infect both neural progenitor cells (NPCs) and mature neurons. A recent study suggested that this virus can replicate within neurons after observing viral particles and endoplasmic reticulum budding in organoids. While, in another study, SARS-CoV-2 viral replication was demonstrated in choroid plexus organoids (CPOs) [8]. This deadly virus also has the ability to use the system of neurons to replicate in the neuroepithelium, which causes inflammation far away and is linked with anosmia and ageusia. In contrast, within the olfactory bulb, endothelial cells have also been categorized with viral proteins and viral RNA transcripts [2,7,9]. After viral infection, the olfactory bulb, thalamus, and brain stem are intensely infected through a trans-synaptic transfer of virus, which instructs the release of chemokines, cytokines, and inflammatory signals immensely to the blood–brain barrier and infect the astrocytes, which cause neuro-inflammation to neuron death, and this induction of excessive inflammation and immune response developed in more neurodegeneration complications. Psychiatric problems, such as depression, sleeping disorders, fatigue, agitation, confusion, inattention, disorientation, and traumatic memories, are unexceptional in patients with COVID-19 infection [3,9,10].

Alzheimer’s disease and dementia both are eminent cognitive impairments after SARS-CoV-2 infection, specifically in critically ill patients [3,11]. To study more about the neuropathogenesis of COVID-19, it is better to understand the histopathological discoveries of the central nervous system. Astrocytes and microglia inflammation’s reactivity is related to its systemic response in the brain. Ischemic lesions, microvascular and peri-vascular hemorrhages were just studied in infected patients [9]. Neuro-radiological and neuro-pathological studies explained the coronavirus effects on brainstems in the central nervous system [12] and pathophysiological pathways of virus in COVID-19 infected patients. The multi-omics data sets have been generated, and pathobiology like Alzheimer’s disease has been studied recently by using an omics critical approach in SARS-CoV-2 infected patients. At the same time, Cerebral venous sinus thrombosis (CVT) is also reported in coronavirus-infected patients [13]. Pathophysiology showed that neurogenic components are participating in respiratory failure in coronavirus diseased persons [11,12].

This toxic virus leads to microvascular damage due to the strong interface between the mechanistic pathway of the virus and the certain neurological syndrome that leads to multiorgan dysfunctions [14]. Neuropilin 1 (NRP1) excites the entry of SARS-CoV-2 in cells, while basigin (BSG) also acts as a receptor to the virus due to its intense articulation in endothelial cells [11], and these cells can be affected by COVID-19 disease [15]. The vascular endothelial glycocalyx is a part of systemic capillary homeostasis care. Still, its role in endothelial damage during COVID-19 is ambiguous [16], while the combined effect of microvascular injury and capillary damage was analyzed in COVID-19 infected patients [17]. More research is necessary for investigating the neuro-invasive pathways in injuring the central nervous system related to edema in the olfactory bulb and coordination in COVID-19 infected patients [2]. This infection acts as an environmental aspect due to protein accumulation in brain that leads to increase of sporadic neurodegeneration in infested inhabitants. This deadly pandemic is a major problem to tackle nowadays, but its post-infection impairments are under discussion and need high intensity to study its sound effects around the globe [18].

The brain is not the chief organ to be infected by coronavirus-2, but the progression of its infection in different adjacent cells in the central nervous system is indeterminate. The brain should be more sensitive to infectivity over respiratory contact. The present review discussed the virologic, molecular, and cellular mechanisms of possible entry routes of SARS-CoV-2 into the brain. It also explained the effects of SARS-CoV-2, its symptoms related to neurons, mechanism of action, potential therapies, present challenges, and future directions for a better understanding.

## 2. Neurological Effects of COVID-19

**Figure 1:**
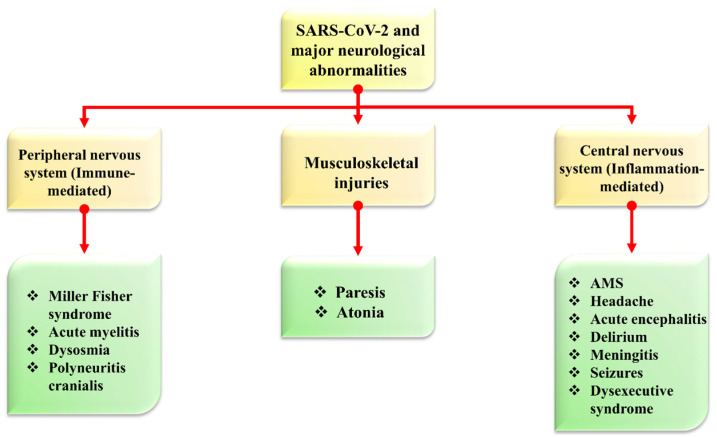
The symptomatic representation of COVID-19 and major neurological abnormalities. The figure is reproduced from Tancheva et al. [22] (Attribution 4.0 International (CC BY 4.0)).

Coronavirus was initially observed as a pathogen responsible for respiratory illness with cough, shortness of breath, fever, muscle pain, headache, sore throat with loss of taste and smell. These symptoms were declared by the center for disease control (CDC). Still, this virus was later considered a pathogen that negatively impacted multiple organs. Its heterogeneity showed more neurological disorders, indicating its RNA in the cerebrospinal fluid of its infected patients in acute and long-term phases [19]. SARS-CoV-2 may directly impact the white and grey matter of the brain and spine with demyelinating lesions and symptoms of seizures, ataxia, dizziness, and cerebrovascular illness. French and Turkish scientists also reported neurological symptoms during coronavirus infection in patients with agitation, delirium, ischemic stroke, and encephalopathy, along with anosmia and dysgeusia, while headache is the most common manifestation (see Figure 1) [20]. This viral infection of the respiratory tract has the strength to cause psychiatric and neurological problems because COVID-19 is neurotropic in nature and has the strength to enter into the central nervous system, while antibodies of COVID-19 have been observed in deceased patient’s cerebrospinal fluid. It is essential to consider COVID-19 as a neuropsychiatric manifestation of long-term complications [21].

Another study observed that obscured monocular vision, dysphoria, vomiting, deliria, coma, brain herniation, loss of consciousness, up-rolling eyeballs, vomiting, four-limb twitching, confusion, and seizures are common in patients infected with the novel coronavirus [19]. However, the neurotropic and neuro-invasive symptoms were observed since the first case of coronavirus in human in 2003 [23]. A few neurological manifestations due to novel coronavirus are mentioned in Table 1.

## 3. Methods and Criteria for Identifying COVID-19 in Brain Cells

Coronavirus enters the brain by retrograde transport via blood circulation access; sensory nerve endings within these regions (cranial olfactory) and angiotensin-converting enzyme-2 are the distinct pathways to enter the brain, especially in the central nervous system. The presence of this SARS-CoV-2 in the brain can be analyzed by systematic analysis of peripheral tissues and brain autopsy. Nucleic acid testing is a common technique for SARS-CoV-2 detection, and it is not valuable for false-positive results in most cases due to sampling or human error [49]. The viral RNA from COVID-19 patients can be detected by RNA scope in situ hybridization in paraffin and formalin-fixed samples. Immunostaining and ultra-structural analysis are used to separate the RNA. RT-PCR is also a detectable method and provides results with high accuracy [50]. Brain autopsies and neuroimaging are also demonstrative ways to identify its presence in the brain [51]. One-step real-time (rRT-PCR) is another method that provides quantitative information to observe its existence. Every technique for coronavirus detection has its drawback and needs more research and work to make these techniques more beneficial. These processes can timely diagnose its presence and reduce its spread and mortality rate [49].

## 4. Routes for Entry of COVID-19 into the Brain and Neurological Manifestations

COVID-19 can spread throughout the central nervous system, impacting the brain and spinal cord, and neurological symptoms could explain this in people infected with long-term infection [52]. This novel virus enters into the brain and tissues through multiple ways, but there is a semi-permeable membrane, a blood–brain barrier that allows only selective nutrients, pathogens, and toxins to flow to and from the brain. This barrier strictly controls the neuronal microenvironment while maintaining neurons’ normal function. However, in the case of other diseases, this blood–brain barrier breaks down, and this dysfunction leads to the cause of infection, that is neurological deficits [53].

**Figure 2:**
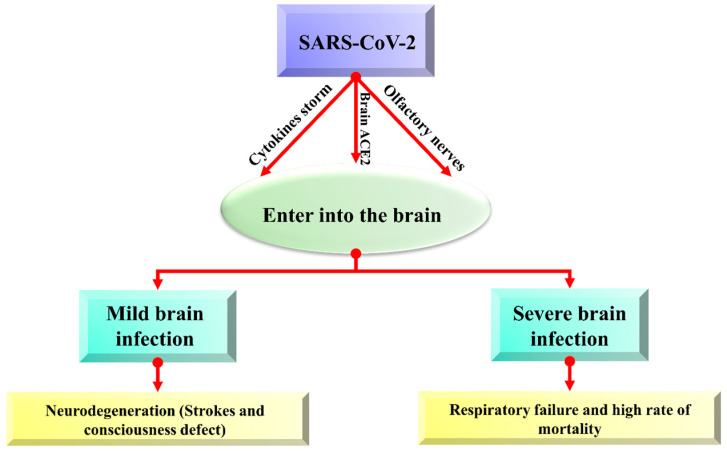
The overview of possible entry routes of SARS-CoV-2 in the brain.

Brain cells can be harmed due to the triggered production of immune molecules and reduced blood flow to the brain. Sometimes, this contagious and deadly coronavirus directly enters the brain and affects its functionality. Viruses may spread to the brain via the lining of the nasal cavity in olfactory mucosa that borders the brain and infects astrocytes over other cells, and neurological symptoms can be described in the brain [54]. About 0.04% of long-term SARS infected patients showed symptoms of the affected central nervous system, and this problem is becoming scarier, while only 0.2% of neurological symptoms were observed with MERS [55]. There are possibly three main ways (olfactory bulb, ACE pathway, and cytokine storm) that coronavirus follows to enter the brain, as shown in Figure 2.

### 4.1. Coronavirus Enters the Brain via ACE2 Pathway

**Figure 3:**
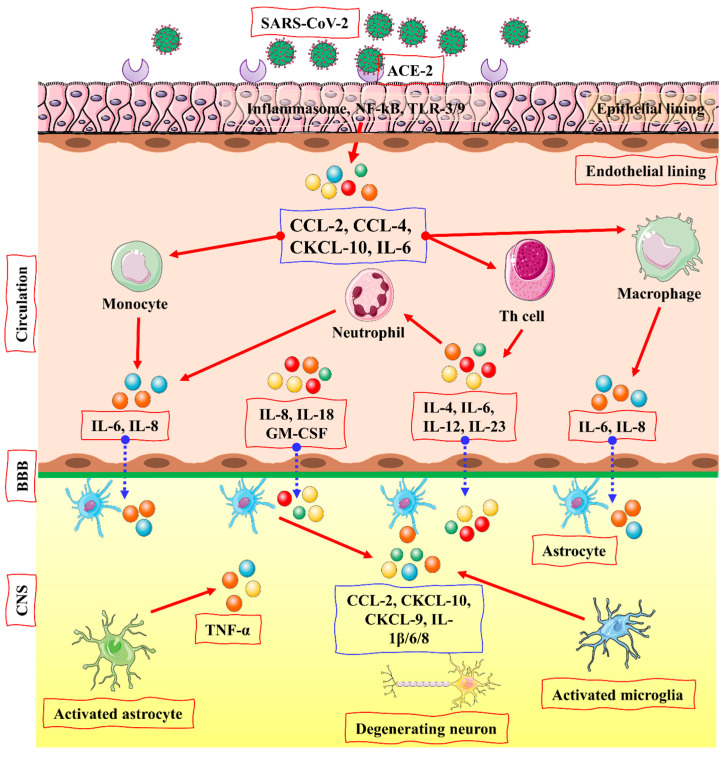
The entry of SARS-CoV-2 in the brain via ACE2 pathway by activation of the cytokine storm. TLR or NF-κB signaling may activate the pro-inflammatory pathway after viral attachment and penetration into epithelial cells via ACE-2 receptor, followed by the development of an inflammasome. CCL-2, CCL-4, CXCL-10, and IL-6 are among the pro-inflammatory cytokines and chemokines generated as a result of this self-defense process. Immune cells, such as monocytes, macrophages, T cells, and neutrophils, are drawn to the infection site by these proteins. A pro-inflammatory feedback loop is created when T lymphocytes produce TNF- β, IL-6, IL-4, IL-12, and IL-23, increasing immune cell accumulation. CNS immune cells, astrocytes and microglia may be activated by these cytokines. IL-1, IL-6, TNF- α, and IL-8 are released as a result of the activated microglia and astrocytes. Several CNS disease-related illnesses are linked with elevated levels of these inflammatory cytokines. The figure is reproduced from Jakhmola et al. [63] after permission from Springer Nature (License No. 5337121441597).

The COVID-19 virus enters the brain via angiotensin-converting enzyme (ACE)-2 receptors present in the CNS and is particularly expressed in the nasal mucosa [56]. A transmembrane protein ACE-2 (Angiotensin-converting enzyme-2) is typically recognized for its carboxypeptidase activity and its physiological role in the renin-angiotensin system (RAS) [57]. ACE-2 (Angiotensin-converting enzyme-2) has an ability to express in many tissues of the brain, and novel coronavirus can directly interact with this enzyme in the capillary endothelium due to which the blood–brain barrier is devastated, which enhances virus entry into the brain and affect the central nervous system, because ACE-2 can express throughout the brain. Viral mRNA also interacts with ACE-2 in COVID-19 infected patients. Increased neurological infections are due to a novel coronavirus that is promoted to enter the central nervous system because of the abundance of ACE-2 enzymes in the brain [58,59]. Viral spike protein interacts with the ACE-2 receptor, an important regulator of the renin-angiotensin system (RAS). Its expression has also been studied in the retina, cerebellum, cerebrum, and olfactory mucosa [60]. The virus enters the brain via other receptor cells, too, like neuropilin-1 (NRP1), basigin (BSG; CD147), cathepsin L (CTSL), and serine protease 2 and 4 (TMPRSS2/4) (see Figure 3) [60,61,62].

### 4.2. Coronavirus Entry into the Brain via Olfactory Pathway

**Figure 4:**
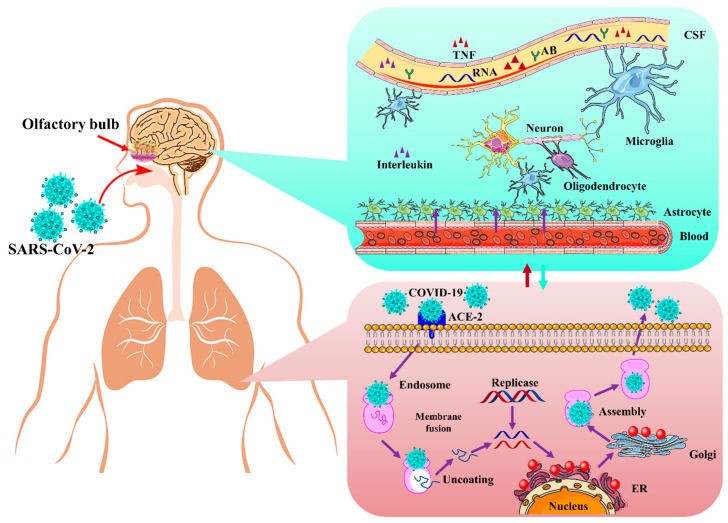
The mechanism of action of SARS-CoV-2 infection in the brain via an olfactory pathway. There are many routes in which SARS-CoV-2 may get into the neural system, including the olfactory nerve. TNF: tumor necrosis factor; ACE2: angiotensin-converting enzyme 2; ER: endoplasmic reticulum; Ab: antibody; CSF: cerebrospinal fluid. The figure is reproduced from Tancheva et al. [22] (Attribution 4.0 International (CC BY 4.0)).

The presence of a high amount of virus in the nasal epithelium suggests that novel coronavirus can travel to the brain through olfactory nerves. Such viruses that can invade neurons can bind with olfactory receptor neurons [64]. nCoV-19 migrates from the cribriform plate by following the trigeminal pathway and penetrating the olfactory mucosa, which causes smell loss and may enter the brain [10]. The virus attaches to olfactory receptors, enters the neuroepithelium, then spreads in the brain stem, thalamus, and medulla oblongata and causes serious disorders. Postmortem reports also provided evidence of its presence in neural endothelial cells in frontal lobe tissues (see Figure 4) [65].

### 4.3. Coronavirus Entry in the Brain via Cytokine Storm

Cerebrospinal fluid and the blood–brain barrier help in creating a balanced environment to protect the brain from viruses and other pathogens, but these can enter via peripheral nerves and olfactory sensory neurons. SARS-CoV-2 trigger pro-inflammatory microglia phenotype (M1 phenotype) that activate proinflammatory cytokines and enhance neurodegenerative disorders [66]. Activating proinflammatory cytokines enhances neutrophil activation, resulting in the characteristic cytokine storm. Interleukin (IL)-1, IL-6, IL-12, interferon (IFN) γ, and tumor necrosis factor (TNF) α, which mainly targets the lung tissue, are also released along with proinflammatory cytokines. Damaged neuroepithelium cells cause inflammation and activate cytokine storms that damage neurons and neurological disorders. Cytokine storm is also associated with immune effector cell-associated neurotoxicity syndrome (ICANS) and other encephalopathies associated with cytokine storm [67].

**Figure 5:**
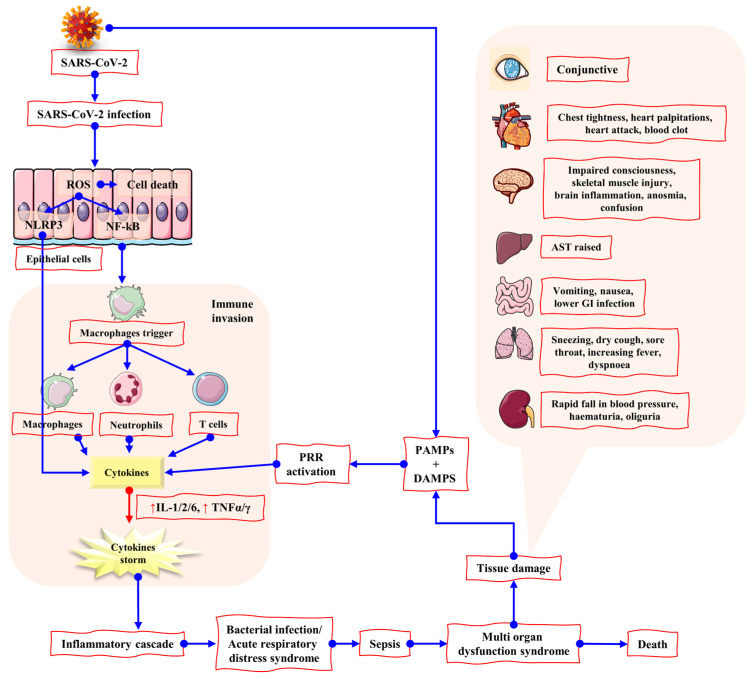
The mechanism of action of tissue damage triggered by cytokine storm after SARS-CoV-2 infection. SARS-CoV 2 infection may lead to a hyperinflammatory immune response in which reactive oxygen species (ROS) generation by epithelial cells can lead to cell death. NLRP3 and NF-κB production may also be stimulated by ROS, resulting in elevated cytokine levels and the cytokine storm. As a result, the body’s immune system is invaded, resulting in potentially fatal disorders, including ARDS and sepsis. MODS has been demonstrated to influence a variety of organs and their related symptoms. Due to the large concentration of ACE2 receptors in the GI tract, it is more susceptible to infection by COVID-19. ROS: reactive oxygen species; PRR: pattern recognition receptors; DAMPs: damage-associated molecular patterns; PAMPs: pathogen-associated molecular patterns; NLRP3: (NOD)-like receptor protein-3 inflammasome. The figure is reproduced from Bhaskar et al. [69] (Attribution 4.0 International (CC BY 4.0)).

Increased levels of cytokines, including Macrophage-Colony Stimulating Factor (M-CSF), interferon γ-induced protein (IP-10), and Macrophage Inflammatory Protein 1-α (MIP1-α). The activated cytokine storm results in a hyperactive immune response that leads to inflammation and inflammatory cytokines, followed by a delayed production of interferons (IFN). The released cytokines stimulate the neutrophils that triggers inflammation when the virus replicates within the macrophages resulting in apoptosis (see Figure 5) [68].

## 5. How Does COVID-19 Help to Block the Blood Vessel?

Blood clotting, another symptom due to COVID-19, is observed, and according to a report, heart attacks and stroke are most observed in novel viral infected patients. Moreover, 40% of deaths are due to cardiovascular complications in infected persons. Endothelial cells that are the inner linings of the walls of blood vessels protecting the cardiovascular system release specific protein that influences blood clotting and response, but these endothelial cells are also infected by the SARS-CoV-2 virus [70]. A prothrombotic state due to elevated fibrinogen level and fibrin degradation in the blood is also a pathophysiological feature of COVID-19, but patients show minor changes in prothrombin and activated partial thromboplastin time, activated protein C levels, and at the time of platelet count. COVID-19 infection with antithrombin activity may cause a damaging situation. Thrombosis and disseminated intravascular clotting are common in SARS-CoV-2 infections [71]. Increased level of fibrinogen, presence of lupus anticoagulant, and high-titer antiphospholipid antibodies that are linked with COVID-19 for its from top to toe risk of coagulopathy [72]. Cerebral venous thrombosis is also linked with a greater risk of COVID-19 [73]. A smooth surface of the endothelium prevents thrombosis and attachment of clotting proteins, so blood flows smoothly in blood vessels. However, endothelial walls are disrupted due to high ACE2 in the endothelium of blood vessels that facilitates the high-affinity binding of SARS-CoV-2 by using spike protein and internal infection/injury on the inner side of the vascular wall blood vessels. Internal blood clots formed, and coagulation activated due to this viral injury [74].

## 6. Invasion and Histopathology of SARS-CoV-2 in Brain

SARS-CoV-2 can induce in the brain. This ability of the pathogen was proved in previous studies of coronaviruses after analyzing autopsy and clinical-based reports that proved its invasion of the brain. Recently, an extensive survey has been conducted to study SARS-CoV-2 in humans, and for this purpose, sixteen brain parts of twenty dead persons from COVID-19 were used for RT-PCR detection of neuroanatomy. Two of these were observed with severe neuropathy, while others had non-specific histopathology. Of the subjects, 20% were detected with novel coronavirus RNA in the entorhinal area, dorsal medulla, olfactory bulb, and leptomeninges [75].

In another study, after sampling eighteen subjects under reverse transcription-quantitative polymerase chain reaction (RT-qPCR) study, there was limited evidence that viral RNA and nucleocapsid proteins of SARS-CoV-2 were harmful to neurons, immune cells, glia, and endothelium [76]. Autopsy-based study of patients with neurological abnormalities showed brain fog but not such chronic inflammation and changes in neurons experienced with viral RNA. However, in a recent study, viral RNA may enter parenchyma during hemorrhage and in brain blood circulations, leading to severe neurological abnormalities [77].

Brain strokes, hemorrhage, and encephalomyelitis have also been described in COVID-19-infected patients because the virus can enter the nervous system after binding its spike protein subunit with receptor angiotensin-converting enzyme-2 (ACE-2) and cause severe injuries. The neurochemical activities in the early stages of COVID-19 infection result in neurogenic stress cardiomyopathy [78]. Chronic and acute neurological diseases are present in brain autopsies of COVID-19 infected patients, and low levels of viral RNA have been observed [79]. Bradley et al. [80] studied fourteen subjects who died of COVID-19, and histopathological features with viral infection were documented with lymphocytic myocarditis respiratory and gastrointestinal tract. In another study, forty-one subjects who died of COVID-19 underwent autopsy and neurological examinations of brain regions, and 44% were observed with neurodegenerative disorders [81].

For diagnosis of COVID-19 induced neurological manifestations, the application of neuroimaging techniques used for this purpose. Computed tomography (CT) scans provided images of internal organs, bones, and blood vessels. In contrast, magnetic resonance imaging (MRI) is a unique technique used to monitor structural details of the brain of COVID-19 infected patients and others. Fluid-Attenuated Inversion Recovery (FLAIR) images from patients with COVID-19-related internal brain issues can be analyzed easily. Magnetic resonance spectroscopy (MRS) studies reflect metabolite abnormalities. Multivoxel MRS imaging has also been performed in patients with COVID-19 with white matter disorder, cardiac arrest, and other cases [82]. Few studies of neurological disorders with brain imaging results are described in Table 2.

## 7. Impact of COVID-19 on CNS and PNS

Neurodegeneration and neuroinflammation are the long-term consequences of COVID-19 invasion in central nervous system (CNS) that might be indicated due to loss in olfactory functions and non-motor symptoms alterations [94,95]. Neurons lost in dearth of inflammation lead to neurodegeneration. SARS-CoV-2 exhibits a constant potential of latency in CNS, and its persistency in the brain causes aggravation of certain diseases. Inactivity of COVID-19 neuro-invasion in CNS is still under consideration, and in this regard, the stable estimation of its intuition to overcome its effects is of specific worth [95]. The direct impact of this virus has been confirmed in cerebrospinal fluid. Agitation, misperception, seizures [96], diffused corticospinal tract signs, anxiety and depressed moods are some possible CNS expressions of coronavirus [94]. A widespread ischemic lesion resembling CNS vasculitis that is an exceptional neurological complicated situation of SARS-CoV-2 has been reported [97]. Disruption of water and sodium regulation are the causes of arterial wall rupture that results in angiotensin II receptor dysfunctionality due to viral invasion. This infection provokes cerebrovascular diseases [96]. Neurological damage and CNS inflammation are caused by coronavirus-2 through various mechanisms, including the damage of ACE-2 receptor, cytokine storm/cytokine injury, secondary hypoxia, demyelination, and BBB disruption [98,99]. Encephalopathy, hyposmia, anxiety, meningitis, cerebral bleeding, sinus venous, Alzheimer’s disease, stroke, psychiatric symptoms, and mood swings all are reported in COVID-19 infected patients due to significant involvement of CNS [99].

Coronavirus affects the CNS and the peripheral nervous system (PNS) in various ways. This is all due to the immune system metabolic processes impairment caused by this virus by gaining entry from the binding sites of olfactory nerves and nasal olfactory epithelium, peripheral nervous system effected by the coronavirus [100,101]. Guillain-Barre syndrome (GBS) and secondary hemophagocytic lympho-histiocytosis are the major problems diagnosed in COVID infected patients due to viral infections. GBS–Miller Fisher syndrome overlap and Bickerstaff brainstem encephalitis can convolute the after-effects of COVID-19, but the diagnosis of these, sometimes, may not show the PNS involvement [101]. Pro-inflammatory cytokines are released that may cause peripheral nociceptors hypersensitivity in an acute case of infections while the central sensitivity is observed during chronic stages. In a recent study, Guillain–Barre syndrome (GBS) [96,102], myositis, and isolated cranial neuropathy (ICN) are associated with COVID-19. Pain, such as joint pain, chest pain, headache, ill-defined pain with fatigue, paresthesia, and myalgia, are the most common long-term PNS association symptoms. Complications related to PNS are very rare, but if observed, these may cause more difficult situations in coronavirus-infested patients [102]. GBS, Miller Fisher syndrome, and polyneuritis cranialis along with neuralgia and myalgia have also been reported as indicated by the PNS link [103].

## 8. Autoantibodies in Neurology by COVID-19

To better understand central nervous systems, autoimmunity, autoantibodies to neuronal targets have resulted, and the previous reclassification of diseases resulted from infectious psychogenic reasons. The autoantibodies can develop dysfunction of the brain due to shifts in neurological disorders and give important novel and therapeutic opportunities [104]. Autoantibodies target the brain tissues for hyperexcitability, and autoantibodies describe some aspects of diseases linked with COVID-19 and direct immunotherapies [105]. In a recent report, 10% of patients with COVID-19 infection had antibodies that blocked the activity of type 1 interferons (INF1) and described that autoantibodies against interferons put the high risk of infectious disorders, and COVID-19 has more attention [106]. Autoantibodies neutralize against type 1 interferons and take part in SCV2 pathogenicity by essential anti-viral responses. In another report of COVID-19 infected patients, autoantibodies re-activate that is the feature of systemic autoimmune diseases. Autoantibodies targets the immune related proteins and 5.2% patients of COVID-19 distinguished with autoantibodies [107].

### Pathogenicity of Autoantibodies

Pathogenic autoantibodies are antiphospholipid antibodies that target the phospholipid-binding proteins and phospholipids and cause multiorgan thrombotic damage. Antiphospholipid antibodies include lupus anticoagulant (LA), anticardiolipin (aCL), and prothrombin (PT) and are most frequent in COVID-19 infected patients. Antiphospholipid antibodies (aCL) IgG autoantibody level is an independent risk factor for COVID-19 severity, while the aCL IgM and aβ2GPI are not such kinds of risk. In a case study of 987 COVID-19 patients, 10.2% had to neutralize IgG autoantibodies against type-I IFNs and were absent in other patients with mild infection [108].

**Figure 6:**
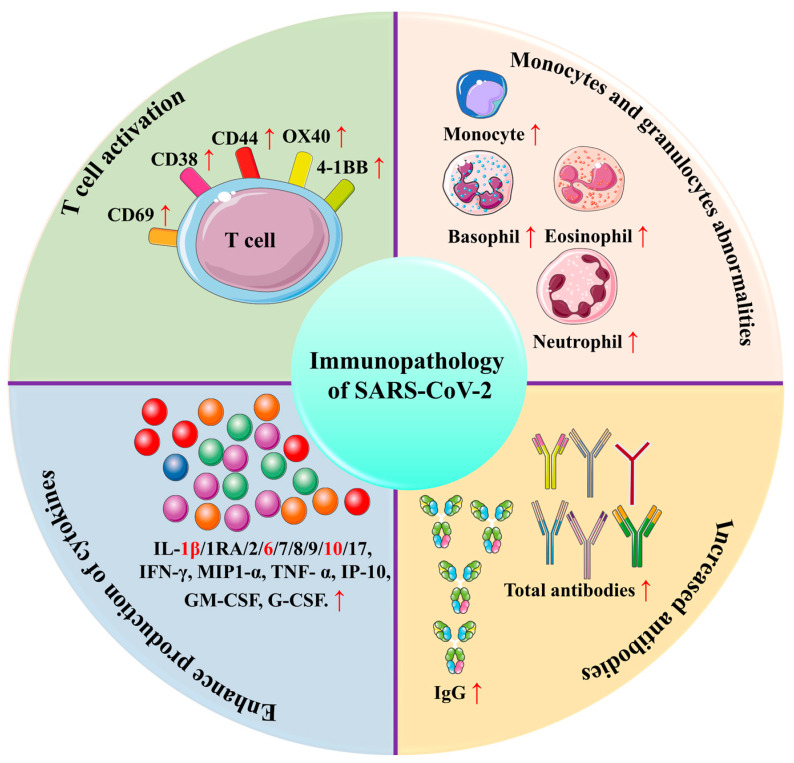
The representation of immunopathology of SARS-CoV-2. Virus-specific T cells from severe cases have a central memory phenotype with high amounts of IFN-γ, TNF-α, and IL-2, while CD38, CD44, and CD69 are highly expressed on CD4^+^ and CD8^+^ T cells. Eosinophil, basophil, and monocyte counts are lower in severe cases, whereas neutrophil counts are more significant. In addition to increased cytokine production, severe COVID-19 has elevated levels of interleukin 1β, interleukin 6, and interleukin 10. The titer of total antibodies is also greater, as are IgG levels. The figure is reproduced from Yang et al. [111] (Attribution 4.0 International (CC BY 4.0)).

Functionally active autoantibodies (AABs) target the G-protein coupled receptors, and many authors consider that autoantibodies are formed during autoimmune processes and be a part of post-COVID-19 diseases. G-protein coupled receptors (GPCR-_f_AABs) were detected in long-term COVID-19 patient’s blood after recovery that targeted the angiotensin II AT1-receptor (AT1-_f_AAB), the β_2_-adrenoceptor (β_2_-_f_AAB), and α_1_-adrenoceptor (α_1_-_f_AAB) [109]. Cellular autoimmunity is linked to neurological symptoms like other viral diseases, and possible mechanisms include molecular mimicry between viral proteins and neuronal autoantigens and late incentive of post-viral immunity [105,108]. People with COVID-19 show a high occurrence of CNS-tissue-linked autoantibodies with immune-targeting autoantibodies and antiphospholipid antibodies. The immunological memory allowed by effector B-cells against self-antigens antibody production in case of diverse CNS tissue targets in the blood–brain barrier and myelin sheath. These CNS tissue autoantibodies resulted in targeted damage with neurodegenerative disease in post-COVID-19 patients (see Figure 6) [110].

## 9. Emerging Immunotherapies against COVID-19

**Figure 7:**
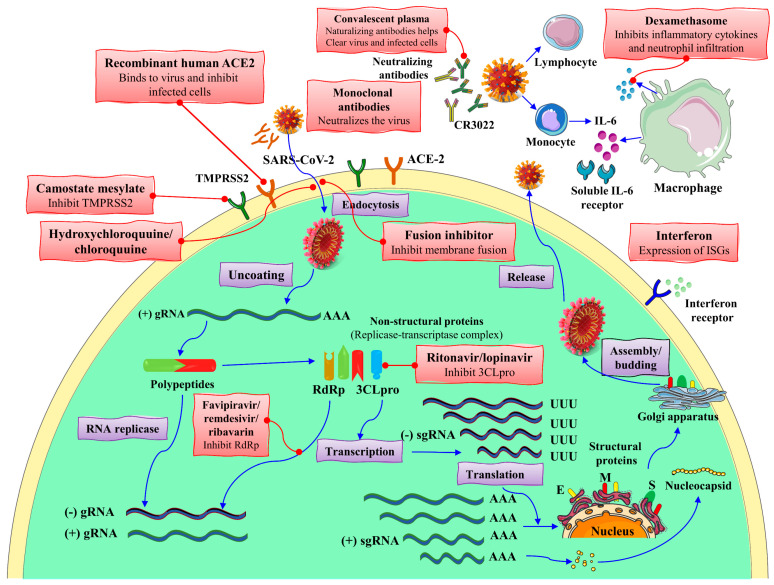
The mechanism of action of SARS-CoV-2 replication and therapeutic approaches. ER: endoplasmic reticulum; HR2P: heptad repeat 2-derived peptides of SARS-CoV-2 spike protein; 3CLpro: 3C-like protease; E: envelope protein; gRNA: genomic RNA; M: membrane protein; ISG: interferon-stimulated gene; RdRp: RNA-dependent RNA polymerases; S: spike protein; sgRNA: subgenomic RNA; TMPRSS2: transmembrane protease serine protease 2. The figure is reproduced from Hu et al. [114] after permission from Nature (License No. 5337130695627).

Immunotherapy is an efficient therapeutic intervention against viral infections. Most immunotherapy attempts have successfully fought against similar COVID-19 viruses, such as SARS-CoV and MERS-CoV. The main methods in this regard include several vaccines and monoclonal antibody candidates [112]. Many studies have been conducted using potential drugs and vaccines to treat and prevent this viral infection. Immunotherapy of COVID-19 includes Natural killer cell therapy (NK therapy), plasma therapy, nano-based therapy, and cytokine immunotherapy are useful during this novel coronavirus worldwide spread (see Figure 7) [113].

The S protein is an acceptable vaccine antigen contender due to its high immunogenicity and ability to make neutralizing antibodies after experiences in the production of SARS-CoV vaccines. Vector, inactivated, and RNA-based SARS-CoV-2 vaccines containing S proteins are being directed worldwide while trials continue [115]. Immodulon, IMM-101, a heat-treated mycobacterium preparation and analogous to BCG (Bacillus Calmette-Guérin) tuberculosis vaccines, may also increase its effects of immuno-oncology to keep protect from COVID-19 effects among cancer in combination with other drugs. Investigators are trying to develop a drug that can protect from cancer and vulnerable side effects of COVID-19 to boost the patient’s immunity (https://www.ajmc.com/view/drug-that-boosts-immunotherapy-studied-as-tool-to-battle-COVID-19-in-patients-with-cancer, accessed on 5 March 2022).

The identification of pathogen-related molecular patterns triggers innate immunity, and this is the first line of defense against a virus [115]. COVID-19 is sensitive to interferon, so it can be used for immunotherapy by treating with IFN-β, which inhibits the replication and transcription of SCV2 [115]. Subcutaneous injections of interferon alpha-2b (INF- α2b) with LPV/r are used to clear viral RNA from COVID-19 patients and require more investigations [116,117,118]. A combination of IFN-α2b and IFN-γ has been described as pharmacodynamics with antiviral activity and is involved in the defense of novel coronavirus. Interferons are glycoproteins with strong antiviral and immunomodulatory properties. Patients received two times a week for two weeks 3.0 million international units (MIU) IFN-α2b and 0.5 MIU IFN-γ (HeberFERON) and lopinavir-ritonavir as a treatment against virus along with CQ of 250 mg that eliminated the virus when observed with only IFN-α2b alone [119]. The common concern is required to treat type 3 (T3 IFN-λ) interferons against viral infection [120]. Type 1 and type 3 interferons are promising anti-SCV2 therapies. Both type I and type III IFNs avoid SARS-CoV-2 in vitro and suggest its potential value of exogenic IFN direction to help viral resistance and prevent disease progression. IFN-λ interferon Lambda has appeared as a capable treatment for COVID-19 and has given a plausible mechanism of action with suppression of IFN activity by SARS-CoV-2. Studies reveal that IFN-λ direction can hinder SCV2 replication [121].

Colchicine, the use as an anti-inflammatory drug in SARS-CoV-2 infection, has been confirmed with its beneficial effects because it reduces the rate of hospitalization ventilation and reduces the risk of mortality [122]. Colchicine inhibits inflammasome signaling and reduces pro-inflammatory cytokines, and it is suggested to use anti-inflammatory action and target inflammasome action as effective therapies [123]. Colchicine possess antiviral properties over inhibition of microtubule polymerization and direction of antioxidative factor productivity [124]. This drug is quickly and well tolerated to reduce the risk of oxygen therapy, hospitalization, and death due to COVID-19 [125]. It also reduces supplemental oxygen therapy [126].

Tocilizumab, an anti–interleukin-6 receptor monoclonal antibody, has been approved for the treatment of multiple inflammatory diseases and seems to improve the patients with COVID-19 infection. Tocilizumab reduced the progression of mechanical ventilation and death, but it did not progress survival [127]. It has been reported as a better outcome in severe COVID-19 patients [128]. Tocilizumab, sarilumab, and siltuximab (IL-6 receptor antagonists) were proposed for COVID-19 treatment [129]. IL-6 receptor blocking agent has been proposed as a treatment for COVID-19 [130]. Tocilizumab efficacy in COVID-19 patients has led to different results [131,132].

## 10. Present Challenges and Future Perspectives

Coronavirus is not only affecting the respiratory centers, but it is more damaging to the brain’s central nervous system and becomes more attention-seeking by a risk factor of increasing cognitive impairment in patients. Alzheimer’s disease and dementia are much more likely to be caused in COVID-19 infected persons and patients already suffering from these disorders are more likely to be affected by a coronavirus. In a recent study, these impairments were shown to be due to neuroinflammation and microvascular damage in the tissue, and single-nuclei levels in the brain imply that SARS-CoV-2 has directly infected the brain [11]. The link and process among these severe impairments need more research to tackle the situation in patients more accurately. To develop the preventative and medicinal measures, an excessive knowledge of its underlying paths via which COVID-19 may cause cognitive impairment is still under more clarification.

Neuropathologic inferences that the RNA transcript of coronavirus in the brainstem and medullary damage in the respiratory center suggest the pathogenesis of COVID-19 transmitted respiratory failure holds a neurogenic constituent. COVID-19 in the vagus nerve indicates the viral transfer between the brainstem and the lungs [12]. More research on this coronavirus traffic needs more understanding and the clarity of its receptor to enter the brain. The brain inflammatory response and hypoxic-ischemic damage, rather than neuronal viral load, are highlighted by clinical investigations in a recent study of COVID-19 infections [4]. More intense research is required in the near future, specifically in terms of potential medicinal examinations about brain damage and response to the deadly virus.

Levels of oxygen and cytokine can affect the neurotransmitters in the brain, while microvascular injury may lead to long-term COVID-19 symptoms that cause chronic health issues, and it is necessary to investigate the microvascular alterations throughout this disease. A dire need to look at the capillary functions in human organs during this pandemic is an urgent requirement of advanced coronavirus research to understand its role in human health. Last, but not least, glycocalyx consists of glycoproteins, covers the vascular endothelium, and plays an essential role in systemic homeostasis maintenance. Its function is clear, but its role in systemic COVID-19 patients in vascular endothelial injury and inflammation is vague and needs more clarification. Additional research is necessary to understand the complications to develop new therapeutic strategies for the growing world in this pandemic.

## 11. Potential Treatment Solutions for Present Challenges and Future Perspectives for Adults COVID-19 Prognosis

The current therapeutic approaches for COVID-associated neurodegeneration and neuroinflammation are primarily based on the improvement of clinical signs. Currently, antiplatelet therapy, constraint-induced therapy, vagus nerve adjunct therapy, and brain microcurrent stimulation therapy have been used to enhance the neuronal adaptation to damage, enhancing their viability in harsh situations and resulting in the best possible blood circulation restoration in the injured arteries [133]. Antiplatelet therapy is based on dipyridamole drug class in COVID-19 patients based on neurodegeneration and neuroinflammation. Dipyridamole inhibits SARS-CoV-2 multiplication, indicating the therapeutic dose of medication may enhance an infected patient’s antiviral response, and showed significant antiaggregant activities, activating anti-viral immunity and greatly enhancing survival. Through various mechanisms, the activated platelets destroy the viral pathogens or aid in their removal from the body by stimulating neutrophil and the macrophage activity, which in turn facilitates platelet aggregates and microthrombi as well as neutrophil extracellular traps formation [134].

Dipyridamole possesses antithrombotic and vasodilator, antioxidant activities, which inhibit neurodegenerative processes and improve neuronal impairment in COVID patients. Dipyridamole also increased the number of circulating platelets and lymphocytes, reduces the D-dimer level, and significantly improved clinical outcomes in COVID patients suffering from neurodegenerative diseases [135]. The TNF-α and proinflammatory cytokines (IL-1, -2) are inhibited by dipyridamole, which also significantly slows down the translocation of nuclear factor NF-B p65 subunit. Dipyridamole offers additional benefits in patients with new viral diseases as a phosphodiesterase inhibitor because it has a vasodilatory effect, raising the level of adenosine, and inhibiting phosphodiesterase in vascular smooth muscle cells [136].

Vagus nerve stimulation (VNS) as an adjunct therapy in COVID-19 attenuates neuroinflammation and plays a significant role in improving the patient clinical outcomes [137]. Non-invasive brain microcurrent stimulation therapy enhances the blood flow and reverses visual impairment, improves blood vessel regulation in peripheral arteries and veins, with all veins that provide the basis for neural reactivation and neurological recovery [138]. Constraint-Induced Therapy (CI Therapy), is currently used to treat patients with traumatic brain injury, restoring lost function and other brain damage in COVID-19 patients. CI therapy consists of a family of treatments that teach the brain to rewire itself following an injury to the brain [139].

Little attention is currently paid to non-drug therapeutic strategies targeting inflammatory and immunological processes that may be useful for reducing the burden of neurological COVID-19-induced complications. There is further need for improving the current treatment status for brain fog attributed to COVID-19. Vagal neurostimulation therapy has a wide field of benefits for patients and should be combined with the best current medical strategies. The development of non-invasive vagal nerve stimulation (t-VNS), a non-pharmacological adjuvant, may help reduce the burden of COVID-19 [140,141].

## 12. Conclusions

Severe acute respiratory syndrome (SARS), a disease caused by coronavirus-2 (CoV-2), belongs to β-coronaviruses is respiratory with pulmonary effects and has a significant impact on the central nervous system as well as peripheral nervous system impairment. This virus has the ability to use the system of neurons to replicate in the neuroepithelium, which causes inflammation far away linked with anosmia and ageusia, while within the olfactory bulb, endothelial cells have also been categorized with viral proteins and viral RNA transcript. COVID-19 enters the brain by involving angiotensin-converting enzyme (ACE)-2 receptors present in the CNS. ACE-2 has the ability to express in many tissues of the brain, and a novel coronavirus can directly interact with this enzyme. SARS-CoV-2 bind to olfactory receptors, enters the neuroepithelium, and then spreads in the brain stem, thalamus, and medulla oblongata. Cytokine storm is associated with immune effector cell-associated neurotoxicity syndrome (ICANS) and other encephalopathies. The pandemic is a major problem to tackle nowadays, but its post-infection impairments are under discussion and need high intensity to study its sound effects around the globe. SARS-CoV-2 is sensitive to interferon, so it can be treated with IFN-β. Interferons have antiviral and immunomodulatory properties to proteins. According to accumulated data, the brains inflammatory response and hypoxic-ischemic damage, rather than neuronal viral load, are highlighted by clinical investigations of COVID-19 infections. However, further study is necessary to understand the complications of SARS-CoV-2 in brain to develop novel therapeutic strategies for the growing world.

## Figures and Tables

**Table 1:** List of neurological disorders caused by SARS-CoV-2 infection.

Disorder	Mean Age (Years)	Onset of Disease	Percentage of Infected Patients	Effect	Treatment/Drug	Reference
Dizziness	39	Shortly after COVID-19	16.8%	Inflammation of the inner ear nerve that connected to the brain	Betahistine, danshenchuandomazine, meclizine, benzodiazepine, steroids, vestibular rehabilitation	[19,24,25,26]
Ischemic stroke and hemorrhage	67.4	In the first week of respiratory symptoms with moderate pulmonary involvement	83.7% stroke and 20.8% hemorrhage	Numbness or weakness in the face, arm, or leg on one side of the body, confusion, difficulty speaking, dizziness, loss of balance, and severe headache	Apixaban 5 mg twice daily, enoxaparin 1 mg/kg every 12 h	[27,28,29,30]
Encephalopathy	66	At the time of documented COVID-19 infection	8.7% while 31.8% in the case study of 509 COVID-19 hospitalized patients	Confusion, non-oriented to time, person, or place, seizures, and sleepiness	High-dose IV steroids, IV immunoglobulin, and immunomodulators (e.g., rituximab)	[31,32,33]
Delirium	77.7	As a sixth primary symptom of coronavirus	28%	Confusion, disorientation, inattention, and cognitive disturbances commonly affect older persons	Haloperidol, melatonin as prophylaxis	[34,35]
Anosmia and Dysguesia	47	Initial symptoms for coronavirus infected patients	47% 54/114 patients and 5.1% anosmia while 5.6% dysgeusia in another study of 214 infected patients	Official symptoms for COVID-19	Caffeine in coffee	[36,37,38,39]
Dysautonomia (also known as secondary COVID-19 infection)	48	Onset 6 weeks following initial COVID-19 symptoms, within the last week of the illness, also seen symptom onset occur within three months of recovery.	50%	Postural lightheadedness and near-syncope, fatigue, activity intolerance, hypertensive response, and orthostatic hypotension	Cefazolin and acebutolol (in case of significant hypertension)	[40,41,42,43]
Microbleed	67.7	Fever, productive cough, myalgias, headache during coronavirus attack	24.4%	Confusion, agitation, and delayed recovery of consciousness	Co-amoxicillin, hydroxychloroquine, piperacillin, tazobactam, azithromycin, lopinavir, ritonavir, levofloxacin, tazobactam	[44,45]
Coma	66	Severe illness due to viral attack	15%	Breathlessness, an erratic heart rate and fatigue, altered mental status, and inability to wakeup off leads to unconsciousness	Modafinil and carbidopa/levodopa, amantadine, aspirin, statin	[46]
Brain herniation, cerebral edema	57	Positive for SARS-CoV 2, fatigue, and fever	3.9%	Hypertension, dyspnea, nausea, vomiting, diarrhea, and multiple juxtacortical hemorrhages (CT scan observation)	Midazolam, low dose norepinephrine	[46]
Cerebral ataxia and myoclonus	59.6	Acute onset within one month of COVID-19	40% ataxia and 46.7% Myoclonus	Spontaneous, action-induced, posture-induced, and mild dysarthria	Methylprednisolone daily for 5 days, clonazepam after 10 days of symptoms, levetiracetam started on day 14	[47]
Seizures	76- and 82-years old patient’s case history	Patients suffering from coronavirus	23% detected by anti-CoV IgM	Convulsive activity and subtle twitching	Antiseizure medication (ASM) therapy, brivaracetam, lacosamide, carbamazepine, phenytoin, phenobarbital, benzodiazepines, valproic acid, vancomycin, meropenem, and Acyclovir for CSF coverage, all drugs should be prescribed cautiously by following doctor’s advice in which patient’s health history is essential	[23,48]

**Table 2:** Case studies of COVID-19 infected patients with neurological manifestations.

Study Case	Average Age (Years)	Manifestation	Reference
Two hundred fourteen (214) patients with the laboratory-confirmed diagnosis of (SARS-CoV-2) infection	58.7	Patients had neurologic manifestations (36.4%), acute cerebrovascular diseases (5.7%), and impaired consciousness (4.8%)	[83]
A retrospective cohort study involving 2054 patients with laboratory-confirmed COVID-19	64	The wide range of neurologic imaging findings in patients with cerebral infarctions (11%), parenchymal hematomas (3.6%) and posterior reversible encephalopathy syndrome (1.1%), 6 cases of cranial nerve abnormalities, 3 patients with a microhemorrhage	[84]
9 patients with a confirmed diagnosis of COVID-19	67.7	Middle cerebellar peduncles (5/9), subcortical regions also affected in patients, micro-bleeding (5/9)	[44]
279 patients hospitalized with COVID-19	__	34% reported memory loss and 28% described impaired concentration, 20% reported cognitive deficits	[85]
219 patients with COVID-19	75.7	Acute ischemic stroke (4.6%) and intracerebral hemorrhage (0.5%)	[86]
153 patients with confirmed COVID-19 cases	71	62% patients with a cerebrovascular event, 74% with ischemic stroke, 12% with an intracerebral hemorrhage, and 1% CNS vasculitis, 31% with altered mental status, 18% patients with encephalitis	[87]
74 patients with a confirmed diagnosis of SARS-CoV-2 infection	64	Altered mental status (53%), fatigue (24%), and headache (18%), patients with ischemic strokes (20%)	[88]
222 COVID-19 patients	65	Encephalopathy (30.2%), acute ischemic cerebrovascular syndrome (25.7%), encephalitis (29.5%) and Guillain-Barre syndrome (6.8%).	[89]
Twenty-seven consecutive patients positive for SARS-CoV-2 who had brain MR imaging	___	26% observed with leukoencephalopathy, mild hypernatremia with an unusual brain MR imaging white matter lesion distribution pattern	[90]
18 patients of COVID-19 with conventional histopathological examination of the brains	50	Fourteen (14) chronic illnesses including diabetes and hypertension, (1) delirium, (5) mild respiratory symptoms, (4) acute respiratory distress syndrome, 2 with pulmonary embolism	[91]
In a retrospective case study of 214 COVID-19 patients	__	Dizziness in 17%, impaired consciousness in 7%, 84% had neurological symptoms that included encephalopathy and associated corticospinal symptoms	[92]
3403 patients with COVID-19, Neuroimaging studies were performed in 167 patients (CT = 172, MRI = 36)	59.7	4.9% had neurological signs, delirium (26%), focal neurology (22%), and altered consciousness (20%), corpus callosum (60%)	[93]

## Data Availability

Not applicable.
